# A European myocardial ^123^I-*m*IBG cross-calibration phantom study

**DOI:** 10.1007/s12350-017-0782-6

**Published:** 2017-01-24

**Authors:** Derk O. Verschure, Edwin Poel, Kenichi Nakajima, Koichi Okuda, Berthe L.F. van Eck-Smit, G. Aernout Somsen, Hein J. Verberne

**Affiliations:** 1 Department of Nuclear Medicine, Academic Medical Center, University of Amsterdam, P.O. Box 22700, 1100 DE Amsterdam, The Netherlands; 2 Department of Cardiology, Zaans Medical Center, Zaandam, The Netherlands; 3 Department of Nuclear Medicine, Kanazawa University Hospital, Kanazawa, Japan; 4 Department of Physics, Kanazawa Medical University, Uchinada, Japan; 5 Cardiology Centers of the Netherlands, Amsterdam, The Netherlands

**Keywords:** ^123^I-*m*IBG scintigraphy, standardization, heart-to-mediastinum ratio, calibration phantom, collimator

## Abstract

**Aim:**

Planar myocardial ^123^I-*meta*-iodobenzylguanidine (^123^I-*m*IBG) scintigraphy is a highly reproducible technique. However, differences in collimator use are one of the most important factors that cause variation among institutions and studies in heart-to-mediastinum (H/M) ratio. Therefore, standardization among various gamma camera-collimator combinations is needed. Previously, a phantom has been developed to cross-calibrate different acquisition conditions in Japan. For further cross-calibration of European myocardial ^123^I-*m*IBG imaging, the aim of this study was to collect ^123^I-*m*IBG data for H/M ratios from common European gamma camera vendors.

**Methods:**

210 experiments were performed in 27 European institutions. Based on these experiments, conversion coefficients for each gamma camera-collimator combination were calculated. An averaged conversion coefficient of 0.88 was used to calculate a standardized H/M ratio.

**Results:**

On average, LE-collimator-derived H/M ratios were significantly lower compared to ME-collimator-derived H/M ratios. The mean conversion coefficients ranged from 0.553 to 0.605 for the LE-collimator group and from 0.824 to 0.895 for the ME-collimator group.

**Conclusion:**

Clinically established H/M ratios can be converted into standardized H/M ratios using cross-calibrated conversion coefficients. This standardization is important for identifying appropriate thresholds for adequate risk stratification. In addition, this cross-calibration enables comparison between different national and international data.

## Introduction

Cardiac ^123^I-*m*IBG scintigraphy, a non-invasive imaging technique to assess cardiac sympathetic activity, has been shown to be of clinical value, especially for the assessment of prognosis, in many cardiac diseases.^1^^–^ 5 The quantification method is essential to differentiate normal and abnormal cardiac sympathetic activity and to distinguish high- and low-risk groups. The heart-to-mediastinum (H/M) ratio is a simple method to correct for background and is highly reproducible with small inter- and intra-observer variation.^6^ However, standardization of acquisition and analysis is needed. The lack of standardization between different institutions is one of the factors that have hampered wide scale clinical implementation of cardiac ^123^I-*m*IBG scintigraphy. International efforts have been made to harmonize and standardize cardiac ^123^I-*m*IBG scintigraphy.^7^ These recommendations include proposals for patient preparation, administered dose of ^123^I-*m*IBG activity (MBq), scanning parameters, and analysis of the acquired data to obtain the most used semi-quantitative parameters [i.e., early and late H/M ratio and ^123^I-*m*IBG washout (WO)].

Collimator choice is one of the most important factors causing variation among institutions and studies.^8^,^9^ In addition to 159 keV photons, ^123^I emits high-energy photons of 529 keV which penetrate the relatively thin septa of low-energy (LE) collimators. This penetration leads to degradation of image quality and ultimately introduces variation in H/M ratios.^10^ Medium-energy (ME) collimators have thicker septa and lower photon penetration compared to LE collimators and therefore have improved image quality and accuracy in myocardial ^123^I-*m*IBG imaging, however, at the expense of spatial resolution.^11^^–^ 13 Consequently, the use of ME collimators is recommended for estimation the H/M ratios.^7^ However, LE collimators are still commonly applied for cardiac ^123^I-*m*IBG scintigraphy because of their wide availability.^7^ In addition, although the nomenclature of collimators is classified into 2 major groups of LE and ME collimators, various types of collimators have been developed depending on the clinical purpose. The variety in the collimator types used has hampered multicentre comparison of cardiac ^123^I-*m*IBG scintigraphy-derived parameters and single-centre results could not easily be extrapolated to other institutions.^14^

In Japan, a phantom for planar cardiac ^123^I-*m*IBG imaging has been developed to cross-calibrate different acquisition conditions.^15^ This phantom has been used to calculate conversion coefficients for different gamma camera-collimator combinations in Japan.^16^ With these conversion coefficients, various conditions can be converted to standard H/M ratios. As an extension of this phantom study, the purpose of this study was to accumulate H/M ratios from common gamma camera vendors in Europe and compare these data with the data from Japan.

## Methods

### Phantom Design and Experiment

**Figure 1:**
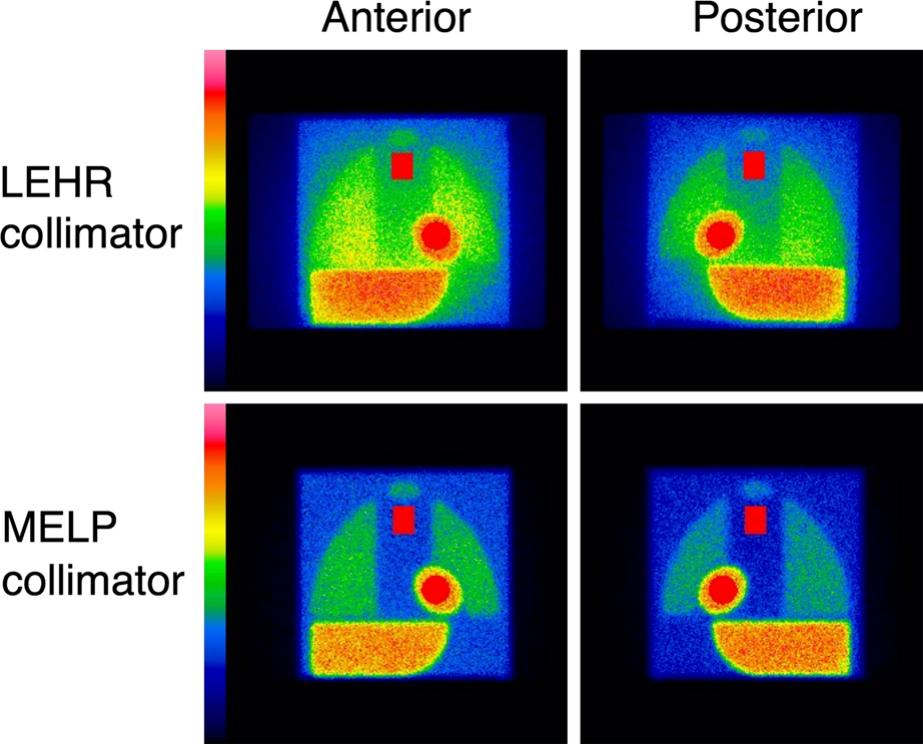
Example of planar ^123^I-*m*IBG images of the phantom in anterior (*left panels*) and posterior (*right panels*) view with Symbia system (Siemens, Erlangen, Germany). Note the difference in image quality due to septal penetration or scatter between low-energy and medium-energy collimators. *LEHR* low energy high resolution; *MELP* medium energy low penetration

A light-weight calibration phantom was used as previously described.^17^ 111 MBq ^123^I was mixed with 4450 ml water to fill the phantom. Since all organ parts were connected as one compartment, no radionuclide concentration adjustment was required for each organ separately. A 3 cm acrylic plate was placed over the phantom to simulate human body attenuation, when imaging was performed. The 256 × 256 matrix images were acquired from the anterior and posterior views for 5 minutes, comparable to clinical planar cardiac ^123^I-*m*IBG imaging (Figure 1). The energy window was centered at 159 keV with a 15% window. The phantom was placed centrally under the gamma camera head with a 5 cm distance between the phantom and collimator surface. The experiments were performed using 210 conditions in 27 institutions in Europe (see ‘‘Appendix’’ for list of all participating institutions).

### Mathematical Reference Value of H/M Ratio

All ^123^I-*m*IBG phantom images were acquired in each participating institution. Data were anonymized and were sent to the Kanazawa University in Japan for central analysis. H/M ratios were mathematically calculated, assuming a linear attenuation coefficient (*μ*) of ^123^I for water as 0.147 cm^−1^. The standard equation for attenuation was used (i.e., exponential of −*μ*x, where x stands for the thickness of the attenuating material). The mathematical calculated reference H/M ratio was corrected for attenuation, while Compton scatter and septal penetration of gamma rays were not included. The reference H/M ratios determined by the structure of the phantom were 2.60 and 3.50 (respectively, anterior and posterior acquisition). Instead of the original phantom used in the Japanese studies, a new light-weight phantom was used in the European study. In the latter phantom, although the dimensions of the phantom were identical, some acrylic parts were made hollow to fill with non-radioactive water. To obtain identical results compared to the original phantom, minor differences in reference values derived from the light-weight phantom were adjusted. The phantom dimensions of the original light-weighted phantom type were measured by CT scan, and the attenuation in the water and acrylics was recalculated. The adjustment of minor difference of phantom design using linear regression line resulted in agreement of conversion coefficients using low energy high resolution (LEHR), low-medium-energy general purpose (LMEGP), and medium energy general purpose (MEGP) collimators.

### Cross Calibration

**Figure 2:**
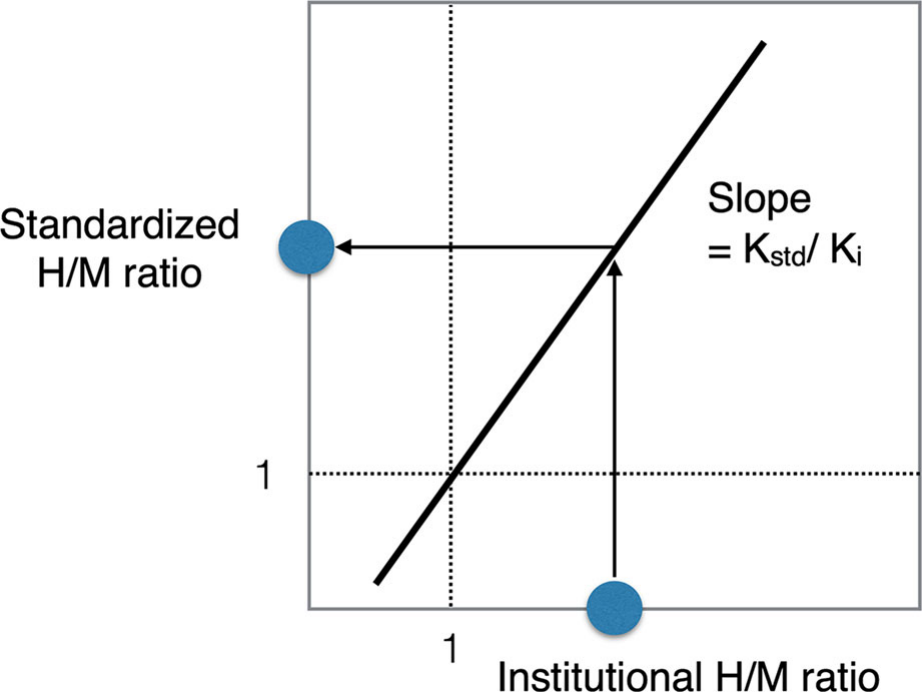
Conversion of H/M ratio from an institutional condition A (H/M ratio_A_) to the standard value (H/M ratio_standard_). The slope of the regression line of the institutional condition, coefficient *K*_i_, and the averaged coefficient of common ME collimators, coefficient *K*_std_ = 0.88, allows for the calculation of a conversion coefficient corrected to a common ME-collimator type

In this study, two H/M ratios (anterior and posterior acquisitions) from each institution were plotted against the reference values (Figure 2). A linear regression equation was calculated using the formula:

$$ {\text{y }} - { 1 } = {\text{ K }}* \, \left( {{\text{x }} - { 1}} \right) $$

(* denotes multiplication), in which the line always passes on the coordinate (1, 1). The coefficient *K*_i_ (i.e., slope of the regression line for each institution) was used to convert the institutional H/M ratios to the reference values (H/M ratio_ref_). In the second step, the H/M ratio_ref_ was converted to a standardized H/M ratio using the *K*_std_. This process can be summarized as:

$$ {\text{Standardized H}}/{\text{M ratio }} - { 1 } = \, K_{{{\text{std}}/}} K_{\text{i}} * \, \left( {{\text{institutional H}}/{\text{M ratio }} - { 1}} \right). $$

The *K*_std_ was 0.88, defined as the average *K* values for typical ME collimators. The rationale for this conversion to the common ME-collimator type is based on the recommendation to use ME collimators.

## Statistics

The data are shown as mean ± standard deviation. Differences among groups were examined by one-way analysis of variance and Student’s *t* test. The linear regression equation of the H/M ratios between two conditions was calculated by the least square method. The statistics software JMP (version 11, SAS Institute Inc., Cary, NC, USA) was used and mathematical calculation was based on Mathematica 10 (Wolfram Research Inc., Champaign, IL, USA).

## Results

210 ^123^I-*m*IBG phantom studies were performed in 27 institutions in Austria, Belgium, the Netherlands, and the United Kingdom including camera vendors of Siemens (*n* = 148), GE (*n* = 44) and Philips (*n =* 18). Collimator types were divided into 2 groups: LE and ME. The LE group included LEHR, general purpose (LEGP), and all-purpose (LEAP) collimators. The ME group included LMEGP, MEGP, and low-penetration (MELP) collimators.

### H/M Ratio Measured in Two Phantom Conditions

**Figure 3:**
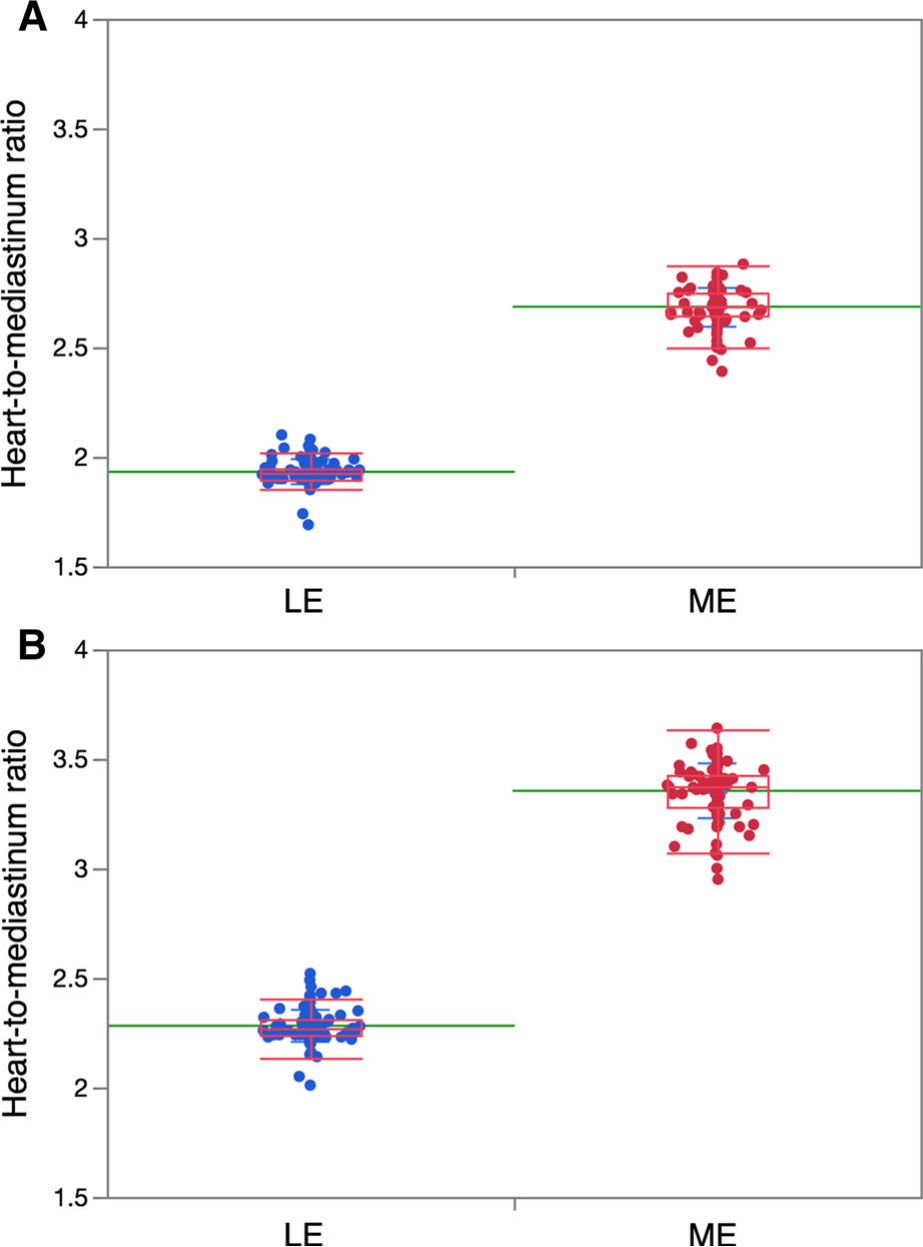
Individual data points and box-whisker plots of H/M ratios using phantoms with the reference H/M ratio of 2.60 (*panel A*) and 3.50 (*panel B*). *Green lines* denote mean values. The *box plot* shows median and the 1st and 3rd quartile, and the ends of the whiskers are ± 1.5 *(interquartile range)

Overall, the LE-collimator group showed lower H/M ratios compared with the ME-collimator group. For the phantom H/M ratio of 2.60, the LE-collimator (*n* = 113)- and ME-collimator (*n* = 97)-derived H/M ratios were 1.932 ± 0.056 and 2.685 ± 0.088, respectively, (*p* = <0.0001). Similarly, for the phantom H/M ratio of 3.50, the LE-collimator- and ME-collimator- derived H/M ratios were 2.281 ± 0.074 and 3.354 ± 0.124, respectively, (*p* < 0.0001) (Figure 3).

### Conversion Coefficients Determined by 2 Data Points

**Figure 4:**
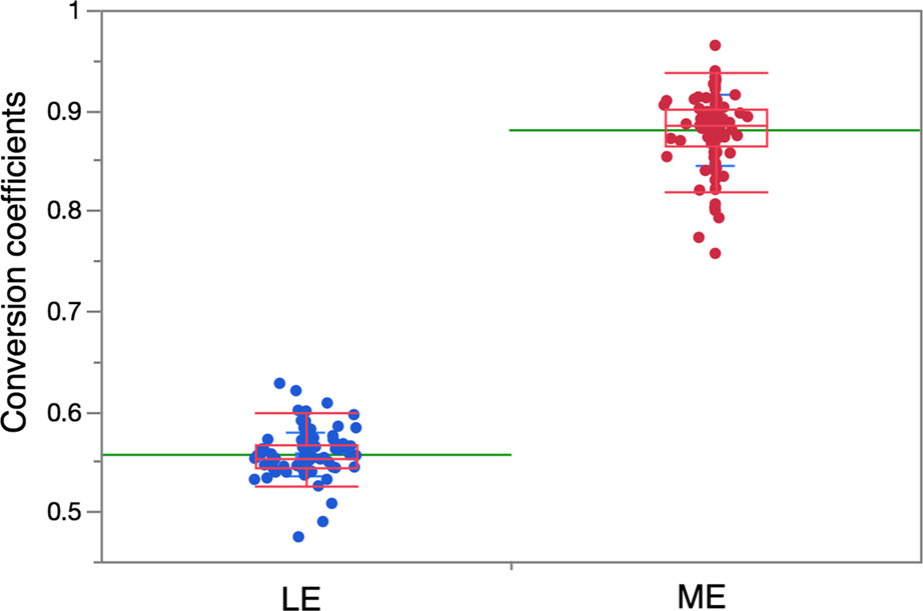
Conversion coefficients to the reference values for the LE- and ME-collimator groups. *Data points* and *box-whisker plots* are also shown. *Green lines* denote mean values. The *box plot* shows median and the 1st and 3rd quartile, and the ends of the whiskers are ± 1.5 *(interquartile range)

**Figure 5:**
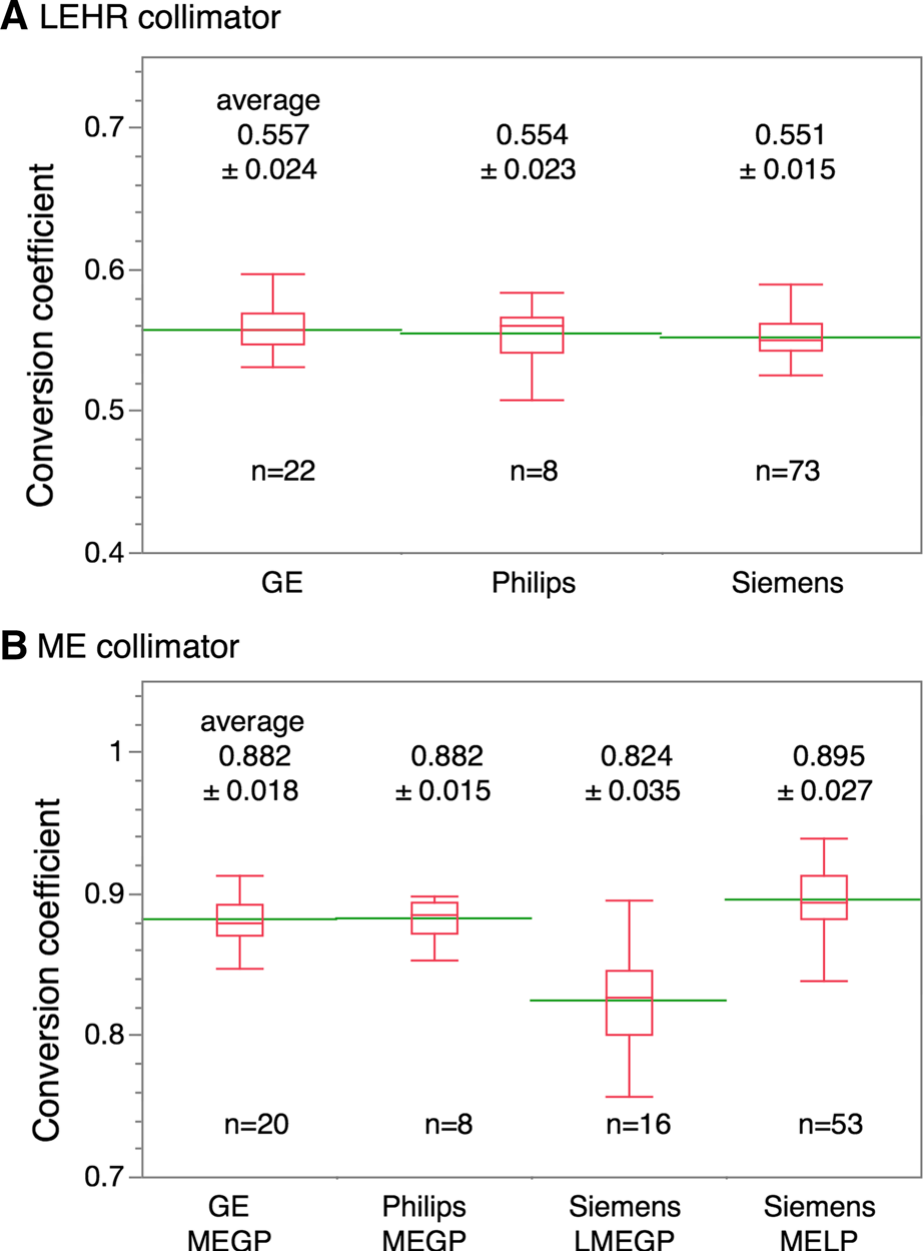
Conversion coefficients of the most common used LE- and ME-collimator types per vendor. *Green lines* denote mean values. The *box plot* shows median and the 1st and 3rd quartile, and the ends of the whiskers are ± 1.5 *(interquartile range)

The conversion coefficients to the reference value are summarized according to the main collimator names: 3 LE sub-groups and 3 ME sub-groups. (Table 1) The average conversion coefficients were 0.553 for LEHR, 0.605 for LEAP, 0.570 for LEGP, 0.824 for LMEGP, and 0.882 for MEGP, and the highest was 0.895 for MELP types. When the conversion coefficients were divided into a LE and a ME group, the average values were, respectively, 0.556 ± 0.021 and 0.880 ± 0.036 (*p* < 0.0001) (Figure 4). Figure 5 shows conversion coefficients of the most common used LE- and ME-collimator types per vendor.

**Table 1:** Conversion coefficient of collimators: European vs. Japanese studies

	Europe			Japan*			*P* values between Europe and Japan
	*N*	Mean	*SD*	*N*	Mean	*SD*	
LEHR	103	0.553	0.018	73	0.552	0.048	0.85
LEGP+LEAP group**	10	0.591	0.024	25	0.648	0.036	<0.0001
LEGP	4	0.570	0.011	17	0.654	0.037	0.0003
LEAP	6	0.605	0.020	2	0.624	0.014	0.15
LMEGP	16	0.824	0.035	46	0.829	0.055	0.74
MEGP+MELP group	81	0.891	0.025	53	0.895	0.061	0.60
MEGP***	28	0.882	0.017	40	0.878	0.054	0.71
MELP	53	0.895	0.027	13	0.950	0.051	<0.0001

*Data from J Nucl Cardiol 2014; 21: 970–978

**LE general-all-purpose collimator is included in Japanese study

*** MEGP, ME general-all purpose, and ME collimators are included in Japanese study

### Comparison Between European and Japanese Conversion Coefficients

Overall, there were no significant differences when the European conversion coefficients of LEHR, LEAP, LMEGP, and MEGP collimators were compared with the Japanese conversion coefficients (Table 1). Only the conversion coefficients for LEGP and MELP collimators differed significantly (*p* < 0.0001). However, when the conversion coefficients for MEGP and MELP were combined, the difference was no longer statistically significant. In contrast when the conversion coefficients for LEGP and LEAP were combined, the statistical significant difference persisted between the European and Japanese data.

## Discussion

These are the results of the first European myocardial ^123^I-*m*IBG cross-calibration phantom study to calculate conversion coefficients for specific individual gamma camera-collimator combinations. The cross-calibration allowed for a conversion of institutional H/M ratios to standardized H/M ratios. These conversion coefficients will facilitate multicentre comparison of myocardial ^123^I-*m*IBG results and enable the extrapolation of the outcome of single- and multicentre studies to other institutions.

The design of collimator septa and apertures has influence on the septal penetration. ME collimators, as recommend by the EANM Cardiovascular Committee,^7^ have thicker septa and lower penetration compared to LE collimators. The difference in collimator types is therefore one of the most important factors that affect variation in H/M ratios. It has been shown that H/M ratios derived from LE collimators are significantly lower compared to those from ME collimators.^8^,^17^ This has been confirmed by the previous cross-calibration phantom study in Japan showing significant underestimation of H/M ratios derived from LE collimators.^16^ As H/M ratios help differentiating high-risk and low-risk groups, this could have clinical implications. However, after correction to standardized H/M ratios, LE and ME collimators showed comparable values.^16^

The present study shows that the conversion coefficients of the LE collimators are lower compared to the ME collimators which is in line with previous phantom studies in Japan (Table 1). The conversion coefficients for most LE- and ME-collimator sub-groups did not show any statistical differences between Europe and Japan. However, there was a significant difference in the LEGP and MELP sub-group.

There are several factors that could explain variation in the LEGP and MELP sub-group between the European and Japanese institutions. Most likely, the small number of LEGP and MELP collimators may have resulted in a limited statistical power. In addition, these differences may be explained by small differences in the phantom used. In contrast to the original designed phantom used in Japan, a light-weight phantom was used for the current European study. After careful examination of both cross-calibration phantoms by CT scanner (Symbia T6/16, Siemens. Erlangen, Germany), under the same conditions (120 mAs and 130 kV), there was a small difference of <1 mm of the ^123^I-*m*IBG compartment between the conventional and light-weight types. Furthermore, one could expect minor differences between Japanese and European camera combinations due to differences in the design of the collimator septa and apertures and gamma camera crystals. However, we have confirmed with the manufacturers that both collimators and gamma cameras used are manufactured identical for Europe and Japan. The differences between LEAP and MELP may also be explained by small variations in acquisition. In Europe and Japan, both energy windows of 15% and 20% have been used according to local protocol. The acquisition time ranged from 3 to 10 minutes in Japan and was 5 minutes in Europe. The distance from collimator to phantom was the same in Europe and Japan. Moreover, although ^123^I was manufactured by different companies in Japan (FUJIFILM RI Pharma, Tokyo, Japan) and Europe (GE Healthcare, Eindhoven, The Netherlands), both products showed no contamination of other isotopes. Finally, there might be a difference between used acrylics of the original phantom and water in some compartments of the light-weight phantom. Although both water and the used acrylics have an almost identical decay coefficient with a very similar scatter pattern, this minor difference in phantom design may explain the found differences in coefficient values. In summary although there is no variation between Europe and Japan for most LE and ME groups, there is a minimal difference in the LEAP and MELP collimator group. As shown, there is a variety of possible explanations for this small difference. However, except for the relative small number of experiments, the above mentioned factors are true for all comparisons between the European and Japanese data. Hence, if valid, these factors would have caused also differences between the other collimators groups. Therefore, this difference is most likely driven for the largest part by the relative small number of experiments. Of course variation in phantom design, variation in energy windows, and variation in acquisition time may have also contributed.

Our study has some limitations. Compared to the Japanese cross-calibration study, we only used 2 (2.60 and 3.50) instead of 4 (1.35, 1.80, 2.60, and 3.50) references H/M ratios. However, conversion coefficients from 2 data points were nearly identical to those 4 data points.^16^ In addition, this cross-calibration method only corrects for high-energy photons coming from liver and lungs, which are the most important contributors of counts overestimation in the mediastinum and heart.^8^ However, this method does not correct for high-energy photons coming other organs like kidney and bladder. In addition, it is important to realize that phantom data are only an approximation of the clinical setting. Therefore, the validity of the conversion coefficients is not guaranteed.

In conclusion, differences in gamma camera-collimator combinations can be corrected to standardize ME-collimator values with the use of a cross-calibration phantom. This method can readily be applied, reducing variation in outcome measures and thereby further the clinical role of myocardial ^123^I-*m*IBG scintigraphy.

## New Knowledge Gained

Standardization of H/M ratios has impact on patient management. Most importantly, standardization of H/M ratio allows for the development of a universal prognostic threshold. This could be established by reanalyzing databases from a number of ^123^I-*m*IBG studies previously published. In addition, future multicentre studies should aim for the use of a standardized H/M ratio, overcoming the impact of gamma camera and collimator differences. Finally, to further stress the importance of standardized H/M ratios, standardized values are essential in risk models for cardiac mortality.^18^

## Appendix

Participating institutions included (alphabetical order):

Academic Medical Center (Amsterdam, the Netherlands),

Amphia ziekenhuis (Breda, the Netherlands),

AZ Groeninge (Kortijk, Belgium),

AZ Maria Middelares (Gent, Belgium),

AZ Sint Jan (Brugge, Belgium),

Diakonessenhuis (Utrecht, the Netherlands),

Erasmus Medical Center (Rotterdam, the Netherlands),

Gelderse Vallei (Ede, the Netherlands),

Groene Hart ziekenhuis (Gouda, the Netherlands),

Jeroen Bosch Ziekenhuis (Den Bosch, the Netherlands),

Leids University Medical Center (Leiden, the Netherlands),

Medical Center Leeuwarden (Leeuwarden, the Netherlands),

Onze Lieve Vrouw (Aalst, Belgium),

OLVG oost (Amsterdam, the Netherlands),

OLVG west (Amsterdam, the Netherlands),

Noordwest ziekenhuisgroep (Alkmaar, the Netherlands),

Sint Augustinus (Wilrijk, Belgium),

Spaarne Gasthuis (Hoofddorp, the Netherlands),

Spaarne Gasthuis (Haarlem, the Netherlands),

University Hospital of North Durham (Durham, United Kingdom),

University Medical Center Groningen (Groningen, the Netherlands),

University Medical Center Utrecht (Utrecht, the Netherlands),

UZ Antwerpen (Edegem, Belgium),

UZ Brussel (Brussel, Belgium),

UZ Leuven (Leuven, Belgium)

Wilhelminenspital (Vienna, Austria),

Zuyderland Medical Center (Heerlen, the Netherlands)

## Abbreviations

^123^I-*m*IBG: ^123^I-*meta*-iodobenzylguanidine
H/M ratio: Heart-to-mediastinum ratio
LE: Low energy
LEAP: Low energy all-purpose
LEHR: Low energy high resolution
LMEGP: Low-medium-energy general purpose
ME: Medium energy
MEGP: Medium energy general purpose
MELP: Medium energy low penetration
WO: Washout
